# *catena*-Poly[[trimethyl­tin(IV)]-μ-[(*E*)-2-methyl-3-(3-methyl­phen­yl)acrylato-κ^2^ *O*:*O*′]]

**DOI:** 10.1107/S1600536808019533

**Published:** 2008-07-05

**Authors:** Niaz Muhammad, M. Nawaz Tahir, Saqib Ali, Zia-ur-Rehman

**Affiliations:** a Department of Chemistry, Quaid-i-Azam University, Islamabad 45320, Pakistan; b University of Sargodha, Department of Physics, Sagrodha, Pakistan

## Abstract

The title trimethyl­tin(IV) carboxyl­ate, [Sn(CH_3_)_3_(C_11_H_11_O_2_)]_*n*_, is a carboxyl­ate-bridged polymer in which the Sn atom exists in a *trans*-C_3_SnO_2_ trigonal bipyramidal coordination. One Sn—O bond is a covalent bond [2.114 (2) Å], whereas the other is a dative bond [2.607 (2) Å]. The polymeric chain propagates along the *b* axis of the monoclinic unit cell.

## Related literature

For related crystal structures, see: Muhammad *et al.* (2008*a* ▶,*b* ▶); Niaz *et al.* (2008 ▶); Tahir *et al.* (1997*a* ▶,*b* ▶).
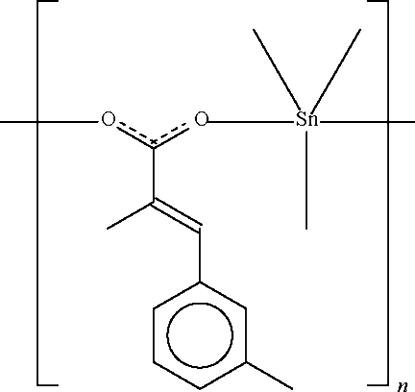


## Experimental

### 

#### Crystal data

[Sn(CH_3_)_3_(C_11_H_11_O_2_)]
*M* *_r_* = 339.01
Monoclinic, 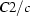

*a* = 12.9530 (6) Å
*b* = 9.8756 (4) Å
*c* = 24.0728 (10) Å
β = 101.301 (2)°
*V* = 3019.7 (2) Å^3^
*Z* = 8
Mo *K*α radiation
μ = 1.68 mm^−1^
*T* = 296 (2) K
0.25 × 0.18 × 0.15 mm

#### Data collection

Bruker Kappa APEXII CCD diffractometer
Absorption correction: multi-scan (*SADABS*; Bruker, 2005 ▶) *T*_min_ = 0.705, *T*_max_ = 0.781
14486 measured reflections
3348 independent reflections
2874 reflections with *I* > 2σ(*I*)
*R*_int_ = 0.023

#### Refinement

*R*[*F* ^2^ > 2σ(*F* ^2^)] = 0.024
*wR*(*F* ^2^) = 0.068
*S* = 1.01
3348 reflections
145 parameters
H-atom parameters constrained
Δρ_max_ = 0.63 e Å^−3^
Δρ_min_ = −0.41 e Å^−3^

### 

Data collection: *APEX2* (Bruker, 2007 ▶); cell refinement: *APEX2*; data reduction: *SAINT* (Bruker, 2007 ▶); program(s) used to solve structure: *SHELXS97* (Sheldrick, 2008 ▶); program(s) used to refine structure: *SHELXL97* (Sheldrick, 2008 ▶); molecular graphics: *ORTEP-3 for Windows* (Farrugia, 1997 ▶); software used to prepare material for publication: *WinGX* (Farrugia, 1999 ▶) and *PLATON* (Spek, 2003 ▶).

## Supplementary Material

Crystal structure: contains datablocks global, I. DOI: 10.1107/S1600536808019533/ng2468sup1.cif

Structure factors: contains datablocks I. DOI: 10.1107/S1600536808019533/ng2468Isup2.hkl

Additional supplementary materials:  crystallographic information; 3D view; checkCIF report

## Figures and Tables

**Table:** 

Sn1—O1	2.1144 (19)
Sn1—C12	2.1126 (17)
Sn1—C13	2.1072 (17)
Sn1—C14	2.1037 (18)
Sn1—O2^i^	2.607 (2)

**Table:** 

O1—Sn1—C12	90.17 (7)
O1—Sn1—C13	97.09 (7)
O1—Sn1—C14	98.56 (7)
O1—Sn1—O2^i^	175.64 (7)
C12—Sn1—C13	114.87 (7)
C12—Sn1—C14	116.04 (7)
C13—Sn1—C14	126.36 (7)

Symmetry code: (i) 
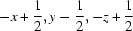
.

**Fig. 1.**
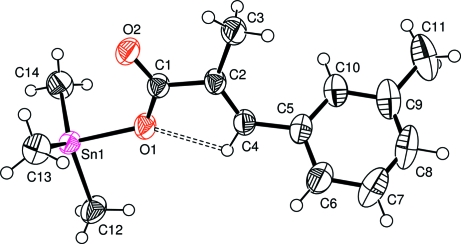
ORTEP drawing of the title compound, (C11H11O2)Sn(CH3)3 with the atom numbering scheme. The thermal ellipsoids are drawn at the 50% probability level. H-atoms are shown by small circles of arbitrary radii. The interamolecular H-bond is shown by dotted lines.

**Fig. 2.**
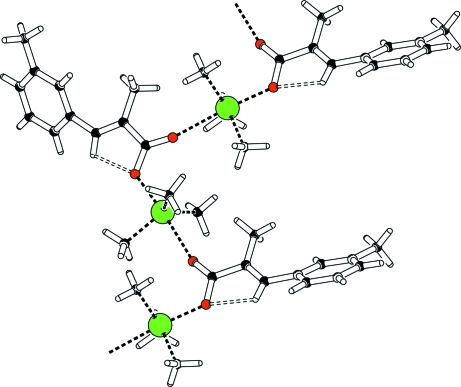
The figure showing the polymeric compound.

## supplementary crystallographic information

### Comment

Organotin compounds have attracted much interest owing to their potential use in industry and agriculture. In the Pharmaceutical industry, a number of dialkyltin carboxylate derivatives are being used as efficient antitumor and anticancer agents. In continuation of synthesizing new ligands having carboxylate groups (Muhammad *et al.*, 2008*a*, Niaz *et al.*, 2008) and their complexation with organotin(IV) (Muhammad *et al.*, 2008*b*), we report the crystal structure of title compound (I).

The title compound (I) (Fig 1.) is the trimethyltin(IV) complex of 3-(3-Methylphenyl)-2-methylacrylate (Muhammad *et al.*, 2008*a*). The crystal structures of (II) {2-[(2,3-Dimethylphenyl)amino]benzoato-*O*:*O*'}trimethyltin(IV) (Tahir *et al.*, 1997*a*) and (III) (Ketoprofenato)trimethyltin(IV) (Tahir *et al.*, 1997*b*) have been reported. As the present complex have similar geometry around Sn-atom, so the bond lengths and bond angles are being compared with (II) and (III). The range of Sn—C [2.1037 (18)- 2.1126 (17) Å] bonds in (I) is reported as [2.106 (3)–2.113 (4) Å] in (II) and 2.106 (6)–2.116 (5) Å, in (III). The range of C—Sn—C [114.87 (7)- 126.36 (7)°] bond angles in (I) is reported as [113.9 (2)°-125.2 (1)°] in (II) and 117.0 (2)°-124.7 (3)°, in (III). Therefore, the C—Sn—C bond angles of trimethyltin moiety is mainly affected due to the change of coordinating ligand. The bond distances for Sn1—O1 [2.1144 (19) Å] and Sn1—O2^i^ [2.607 (2) Å] (symmetry code i = -*x* + 1/2, *y* - 1/2, -*z* + 1/2) have different values compared to (II) and (III). These values in (II) and (III) are [2.153 (2) Å and 2.495 (2) Å] and [2.184 (3) Å and 2.433 (4) Å], respectively. The O1—Sn1—O2^i^ bond angle is 175.64 (7)°, which is larger but not very different from (II) and (III). The dihedral angle between the plane of benzene ring A (C5—C10) and the plane formed by C11/C12/C13 is 76.16 (7)°, whereas it is 7.0 (7)° between O1/C1/O2 and C2/C3/C4. There is a single C—H···O interamolecular H-bond (Table 2, Fig 1.) forming a five-membered ring (O1/C1/C2/C4/H4···O1). There exist π-π-interactions between the centroids of benzene ring [CgA···CgA^iii^: symmetry code iii = 1 - *x*, -*y*, -*z*] and [CgA···CgA^iv^: symmetry code iv = 1 - *x*, 1 - *y*, -*z*]. The perpendicular distance between the centroids for CgA···CgA^iii^ and CgA···CgA^iv^ is 3.488 Å and 3.725 Å, respectively. The compound is polymeric in nature due to the bridging nature of carboxyl group.

### Experimental

The title compound (I), was prepared by the reaction of stoichiometric amounts of the sodium 3-(3-methylphenyl)-2-methylacrylate (0.399 g, 2.02 mmol) and (0.402 g, 2.02 mmol)of trimethyltin(IV)chloride in dry toluene (100 ml). The reaction mixture was refluxed for 8 h and then allowed to stand overnight. The residual sodium salt was removed by filtration and the solvent was evaporated under reduced pressure leaving a solid residue. This was recrystallized from a mixture of chloroform/n-hexane (4:1). The yield was 80%.

### Refinement

H atoms were positioned geometrically, with C-H= 0.93, and 0.96 Å for aromatic and methyl H, and constrained to ride on their parent atoms, with U_iso_(H) = xU_eq_(C), where x = 1.5 for methyl H, and x = 1.2 for other H atoms.

### Crystal data

**Table:**

[Sn(CH_3_)_3_(C_11_H_11_O_2_)]	*F*_000_ = 1360
*M**_r_* = 339.01	*D*_x_ = 1.491 Mg m^−^^3^
Monoclinic, *C*2/*c*	Mo *K*α radiation λ = 0.71073 Å
Hall symbol: -C 2yc	Cell parameters from 3348 reflections
*a* = 12.9530 (6) Å	θ = 2.6–27.1º
*b* = 9.8756 (4) Å	µ = 1.68 mm^−^^1^
*c* = 24.0728 (10) Å	*T* = 296 (2) K
β = 101.301 (2)º	Prismatic, colourless
*V* = 3019.7 (2) Å^3^	0.25 × 0.18 × 0.15 mm
*Z* = 8	

### Data collection

**Table:**

Bruker Kappa APEXII CCD diffractometer	3348 independent reflections
Radiation source: fine-focus sealed tube	2874 reflections with *I* > 2σ(*I*)
Monochromator: graphite	*R*_int_ = 0.023
Detector resolution: 7.5 pixels mm^-1^	θ_max_ = 27.2º
*T* = 296(2) K	θ_min_ = 2.6º
ω sans scans	*h* = −16→15
Absorption correction: multi-scan(SADABS; Bruker, 2005)	*k* = −7→12
*T*_min_ = 0.705, *T*_max_ = 0.781	*l* = −30→30
14486 measured reflections	

### Refinement

**Table:**

Refinement on *F*^2^	Secondary atom site location: difference Fourier map
Least-squares matrix: full	Hydrogen site location: inferred from neighbouring sites
*R*[*F*^2^ > 2σ(*F*^2^)] = 0.024	H-atom parameters constrained
*wR*(*F*^2^) = 0.068	*w* = 1/[σ^2^(*F*_o_^2^) + (0.0374*P*)^2^ + 3.5913*P*] where *P* = (*F*_o_^2^ + 2*F*_c_^2^)/3
*S* = 1.01	(Δ/σ)_max_ = 0.001
3348 reflections	Δρ_max_ = 0.63 e Å^−^^3^
145 parameters	Δρ_min_ = −0.41 e Å^−^^3^
Primary atom site location: structure-invariant direct methods	Extinction correction: none

### Special details

**Table:**

Geometry. Bond distances, angles *etc*. have been calculated using the rounded fractional coordinates. All su's are estimated from the variances of the (full) variance-covariance matrix. The cell e.s.d.'s are taken into account in the estimation of distances, angles and torsion angles
Refinement. Refinement of *F*^2^ against ALL reflections. The weighted *R*-factor *wR* and goodness of fit *S* are based on *F*^2^, conventional *R*-factors *R* are based on *F*, with *F* set to zero for negative *F*^2^. The threshold expression of *F*^2^ > σ(*F*^2^) is used only for calculating *R*-factors(gt) *etc*. and is not relevant to the choice of reflections for refinement. *R*-factors based on *F*^2^ are statistically about twice as large as those based on *F*, and *R*- factors based on ALL data will be even larger.

### Fractional atomic coordinates and isotropic or equivalent isotropic displacement parameters (Å^2^)

**Table:**

	*x*	*y*	*z*	*U*_iso_*/*U*_eq_	
Sn1	0.28429 (1)	−0.16993 (2)	0.22816 (1)	0.0380 (1)	
O1	0.32929 (16)	−0.03474 (18)	0.16907 (8)	0.0498 (6)	
O2	0.26730 (17)	0.1483 (2)	0.20361 (9)	0.0529 (7)	
C1	0.3143 (2)	0.0940 (3)	0.17022 (10)	0.0389 (7)	
C2	0.3576 (2)	0.1747 (2)	0.12708 (11)	0.0401 (8)	
C3	0.3324 (3)	0.3224 (3)	0.12430 (15)	0.0591 (10)	
C4	0.4185 (2)	0.1119 (3)	0.09665 (11)	0.0426 (8)	
C5	0.4713 (2)	0.1667 (3)	0.05244 (12)	0.0482 (9)	
C6	0.5691 (3)	0.1139 (4)	0.04886 (14)	0.0663 (11)	
C7	0.6223 (3)	0.1596 (5)	0.00784 (19)	0.0870 (18)	
C8	0.5750 (3)	0.2546 (5)	−0.03080 (15)	0.0809 (15)	
C9	0.4775 (3)	0.3064 (4)	−0.02935 (13)	0.0663 (11)	
C10	0.4254 (3)	0.2612 (3)	0.01229 (12)	0.0534 (10)	
C11	0.42742 (14)	0.40923 (17)	−0.07152 (7)	0.0932 (18)	
C12	0.35163 (14)	−0.33276 (17)	0.19080 (7)	0.0624 (11)	
C13	0.38060 (14)	−0.08820 (17)	0.30121 (7)	0.0547 (10)	
C14	0.12000 (14)	−0.14855 (17)	0.20410 (7)	0.0586 (10)	
H3A	0.33496	0.35644	0.16190	0.0885*	
H3B	0.26305	0.33584	0.10207	0.0885*	
H3C	0.38277	0.36974	0.10716	0.0885*	
H4	0.42933	0.02018	0.10434	0.0511*	
H6	0.59915	0.04728	0.07422	0.0792*	
H7	0.68900	0.12660	0.00642	0.1042*	
H8	0.61025	0.28430	−0.05866	0.0972*	
H10	0.35872	0.29454	0.01336	0.0640*	
H11A	0.37070	0.45259	−0.05805	0.1396*	
H11B	0.40055	0.36550	−0.10702	0.1396*	
H11C	0.47878	0.47577	−0.07662	0.1396*	
H12A	0.30201	−0.40592	0.18350	0.0935*	
H12B	0.41411	−0.36295	0.21610	0.0935*	
H12C	0.36936	−0.30341	0.15582	0.0935*	
H13A	0.34336	−0.01717	0.31612	0.0820*	
H13B	0.44368	−0.05221	0.29160	0.0820*	
H13C	0.39864	−0.15800	0.32919	0.0820*	
H14A	0.09961	−0.06049	0.21497	0.0879*	
H14B	0.08599	−0.21672	0.22256	0.0879*	
H14C	0.09943	−0.15869	0.16378	0.0879*	

### Atomic displacement parameters (Å^2^)

**Table:**

	*U*^11^	*U*^22^	*U*^33^	*U*^12^	*U*^13^	*U*^23^
Sn1	0.0438 (1)	0.0315 (1)	0.0422 (1)	−0.0025 (1)	0.0169 (1)	−0.0026 (1)
O1	0.0662 (12)	0.0339 (9)	0.0574 (11)	−0.0025 (9)	0.0322 (10)	0.0049 (8)
O2	0.0681 (13)	0.0464 (11)	0.0518 (11)	0.0016 (9)	0.0307 (10)	−0.0030 (9)
C1	0.0445 (14)	0.0348 (12)	0.0393 (12)	−0.0037 (11)	0.0131 (11)	−0.0012 (10)
C2	0.0499 (15)	0.0326 (12)	0.0405 (13)	−0.0067 (11)	0.0152 (11)	0.0005 (10)
C3	0.086 (2)	0.0339 (14)	0.0670 (19)	−0.0010 (14)	0.0383 (18)	0.0022 (13)
C4	0.0512 (15)	0.0386 (13)	0.0409 (13)	−0.0029 (12)	0.0162 (11)	0.0015 (11)
C5	0.0548 (16)	0.0507 (16)	0.0432 (14)	−0.0101 (13)	0.0196 (12)	−0.0023 (12)
C6	0.063 (2)	0.082 (2)	0.0602 (19)	0.0021 (18)	0.0278 (16)	0.0043 (18)
C7	0.068 (2)	0.126 (4)	0.078 (3)	−0.014 (2)	0.041 (2)	−0.008 (3)
C8	0.083 (3)	0.115 (3)	0.0525 (19)	−0.036 (2)	0.0324 (19)	0.002 (2)
C9	0.081 (2)	0.075 (2)	0.0420 (16)	−0.0339 (19)	0.0099 (15)	0.0009 (15)
C10	0.0604 (18)	0.0567 (18)	0.0434 (14)	−0.0169 (14)	0.0111 (13)	0.0004 (13)
C11	0.121 (4)	0.100 (3)	0.053 (2)	−0.040 (3)	0.003 (2)	0.023 (2)
C12	0.085 (2)	0.0397 (15)	0.075 (2)	0.0003 (15)	0.0463 (19)	−0.0039 (14)
C13	0.0519 (16)	0.0603 (18)	0.0517 (16)	−0.0072 (14)	0.0099 (13)	−0.0042 (14)
C14	0.0489 (16)	0.0635 (19)	0.0629 (18)	−0.0053 (14)	0.0095 (14)	0.0069 (15)

### Geometric parameters (Å, °)

**Table:**

Sn1—O1	2.1144 (19)	C3—H3B	0.9600
Sn1—C12	2.1126 (17)	C3—H3C	0.9600
Sn1—C13	2.1072 (17)	C4—H4	0.9300
Sn1—C14	2.1037 (18)	C6—H6	0.9300
Sn1—O2^i^	2.607 (2)	C7—H7	0.9300
O1—C1	1.287 (3)	C8—H8	0.9300
O2—C1	1.223 (3)	C10—H10	0.9300
C1—C2	1.502 (4)	C11—H11A	0.9600
C2—C3	1.493 (4)	C11—H11B	0.9600
C2—C4	1.330 (4)	C11—H11C	0.9600
C4—C5	1.476 (4)	C12—H12A	0.9600
C5—C6	1.388 (5)	C12—H12B	0.9600
C5—C10	1.391 (4)	C12—H12C	0.9600
C6—C7	1.386 (6)	C13—H13A	0.9600
C7—C8	1.378 (6)	C13—H13B	0.9600
C8—C9	1.369 (6)	C13—H13C	0.9600
C9—C10	1.387 (5)	C14—H14A	0.9600
C9—C11	1.492 (4)	C14—H14B	0.9600
C3—H3A	0.9600	C14—H14C	0.9600
			
O1—Sn1—C12	90.17 (7)	C5—C4—H4	115.00
O1—Sn1—C13	97.09 (7)	C5—C6—H6	120.00
O1—Sn1—C14	98.56 (7)	C7—C6—H6	120.00
O1—Sn1—O2^i^	175.64 (7)	C6—C7—H7	120.00
C12—Sn1—C13	114.87 (7)	C8—C7—H7	120.00
C12—Sn1—C14	116.04 (7)	C7—C8—H8	119.00
C13—Sn1—C14	126.36 (7)	C9—C8—H8	119.00
Sn1—O1—C1	123.13 (17)	C5—C10—H10	119.00
Sn1^ii^—O2—C1	159.7 (2)	C9—C10—H10	119.00
O1—C1—O2	122.9 (2)	C9—C11—H11A	109.00
O1—C1—C2	115.5 (2)	C9—C11—H11B	109.00
O2—C1—C2	121.5 (3)	C9—C11—H11C	109.00
C1—C2—C3	116.2 (2)	H11A—C11—H11B	109.00
C1—C2—C4	118.4 (2)	H11A—C11—H11C	109.00
C3—C2—C4	125.4 (3)	H11B—C11—H11C	109.00
C2—C4—C5	129.4 (3)	Sn1—C12—H12A	109.00
C4—C5—C6	117.7 (3)	Sn1—C12—H12B	109.00
C4—C5—C10	123.5 (3)	Sn1—C12—H12C	109.00
C6—C5—C10	118.7 (3)	H12A—C12—H12B	109.00
C5—C6—C7	120.6 (3)	H12A—C12—H12C	109.00
C6—C7—C8	119.1 (4)	H12B—C12—H12C	109.00
C7—C8—C9	121.8 (4)	Sn1—C13—H13A	109.00
C8—C9—C10	118.7 (3)	Sn1—C13—H13B	109.00
C8—C9—C11	121.2 (3)	Sn1—C13—H13C	109.00
C10—C9—C11	120.2 (3)	H13A—C13—H13B	109.00
C5—C10—C9	121.1 (3)	H13A—C13—H13C	109.00
C2—C3—H3A	109.00	H13B—C13—H13C	109.00
C2—C3—H3B	109.00	Sn1—C14—H14A	109.00
C2—C3—H3C	110.00	Sn1—C14—H14B	109.00
H3A—C3—H3B	109.00	Sn1—C14—H14C	109.00
H3A—C3—H3C	110.00	H14A—C14—H14B	109.00
H3B—C3—H3C	109.00	H14A—C14—H14C	109.00
C2—C4—H4	115.00	H14B—C14—H14C	109.00
			
C12—Sn1—O1—C1	−176.6 (2)	C1—C2—C4—C5	−179.4 (3)
C13—Sn1—O1—C1	−61.5 (2)	C3—C2—C4—C5	−2.8 (5)
C14—Sn1—O1—C1	67.0 (2)	C2—C4—C5—C6	145.1 (3)
C12—Sn1—O2^i^—C1^i^	−157.4 (5)	C2—C4—C5—C10	−39.2 (5)
C13—Sn1—O2^i^—C1^i^	87.2 (5)	C4—C5—C6—C7	179.2 (3)
C14—Sn1—O2^i^—C1^i^	−40.4 (5)	C10—C5—C6—C7	3.3 (5)
Sn1—O1—C1—O2	−4.3 (4)	C4—C5—C10—C9	−178.1 (3)
Sn1—O1—C1—C2	176.30 (16)	C6—C5—C10—C9	−2.5 (5)
Sn1^ii^—O2—C1—O1	146.2 (4)	C5—C6—C7—C8	−2.5 (6)
Sn1^ii^—O2—C1—C2	−34.4 (7)	C6—C7—C8—C9	0.9 (7)
O1—C1—C2—C3	174.6 (3)	C7—C8—C9—C10	−0.1 (6)
O1—C1—C2—C4	−8.5 (4)	C7—C8—C9—C11	180.0 (4)
O2—C1—C2—C3	−4.8 (4)	C8—C9—C10—C5	0.9 (5)
O2—C1—C2—C4	172.1 (3)	C11—C9—C10—C5	−179.2 (3)

Symmetry codes: (i) −*x*+1/2, *y*−1/2, −*z*+1/2; (ii) −*x*+1/2, *y*+1/2, −*z*+1/2.

### Hydrogen-bond geometry (Å, °)

**Table:**

*D*—H···*A*	*D*—H	H···*A*	*D*···*A*	*D*—H···*A*
C4—H4···O1	0.9300	2.2800	2.695 (3)	107.00
